# The Effects of Colchicum Dispert and Bone Marrow-Derived Mesenchymal Stem Cell Therapy on Skeletal Muscle Injury in a Rat Aortic Ischemia–Reperfusion Model

**DOI:** 10.3390/jcdd11080251

**Published:** 2024-08-16

**Authors:** Atilla Orhan, Ömer Faruk Çiçek, Bahadır Öztürk, Hakan Akbayrak, Nejat Ünlükal, Hakan Vatansev, Merve Solmaz, Mustafa Büyükateş, Seda Aniç, Fadime Ovalı, Eissa Almaghrebi, Fatma Akat, Hüsamettin Vatansev

**Affiliations:** 1 Department of Cardiovascular Surgery, Medical Faculty, Selçuk University, Konya 42250, Turkey; farux@hotmail.com (Ö.F.Ç.); hakanakbayrak@gmail.com (H.A.); mustafabuyukates@yahoo.com (M.B.); 2 Department of Biochemistry, Medical Faculty, Selçuk University, Konya 42250, Turkey; ozturkbhdr@hotmail.com (B.Ö.); isaahmed9292@gmail.com (E.A.); fatmakat94@gmail.com (F.A.); hvatansev@gmail.com (H.V.); 3 Department of Histology, Medical Faculty, Selçuk University, Konya 42250, Turkey; nejatunlukal@gmail.com (N.Ü.); merveozersolmaz@gmail.com (M.S.); sedaberen17@gmail.com (S.A.); 4 Department of Food Processing, Meram Vocational School, Necmettin Erbakan University, Konya 42092, Turkey; hakanvatansev@gmail.com; 5 Department of Medical Biochemistry, Institute of Health Sciences, Selçuk University, Konya 42250, Turkey; ovali.fadime@gmail.com

**Keywords:** colchicine, ischemia–reperfusion injury, mesenchymal stem cell, peripheral arterial disease, skeletal muscle

## Abstract

Background: Abdominal aortic aneurysms and peripheral artery disease pose significant health risks, ranking third after heart attacks and cerebral strokes. Surgical interventions often involve temporary aortic clamping, leading to ischemia–reperfusion injury and tissue damage. Colchicine and mesenchymal stem cells have shown promise, individually, in mitigating ischemia–reperfusion injury, but their combined effects remain understudied. Methods: This study utilized 42 male Wistar rats, divided into six groups: Control, Sham, Ischemia–Reperfusion, Colchicine, Mesenchymal stem cell, and Mix (colchicine and mesenchymal stem cell). The ischemia–reperfusion model involved clamping the abdominal aorta for 60 min, followed by 120 min of reperfusion. Colchicine and mesenchymal stem cell treatments were administered as pre- and post-ischemia interventions, respectively. Mesenchymal stem cells were cultured, characterized by flow cytometry, and verified for specific surface antigens. Blood and tissue samples were analyzed for oxidative stress markers, nitric oxide metabolites, and apoptosis using TUNEL. Results: There were significant differences between the groups in terms of the serum total antioxidant capacity (*p* < 0.001) and inflammation markers (ischemia-modified albumin, *p* = 0.020). The combined therapy group (Mix) exhibited the lowest inflammation levels. Arginine levels also showed significant variation (*p* = 0.028), confirming the ischemia–reperfusion injury model. In muscle tissues, the total antioxidant capacity (*p* = 0.022), symmetric dimethylarginine, and citrulline levels (*p* < 0.05) indicated nitric oxide metabolism. Apoptosis was notably high in the ischemia–reperfusion injury group as anticipated. It appeared to be reduced by colchicine, mesenchymal stem cells, and their combination, with the most significant decrease observed in the Mix group (*p* < 0.001). Conclusions: This study highlights the potential of using combined colchicine and mesenchymal stem cell therapy to reduce muscle damage caused by ischemia–reperfusion injury. Further research is needed to understand the underlying mechanisms and confirm the clinical significance of this approach in treating extremity ischemia–reperfusion injuries.

## 1. Introduction

Abdominal aortic aneurysms and peripheral artery disease are prevalent health concerns that can lead to significant mortality and morbidity, ranking third after heart attacks and cerebral strokes. Surgical interventions for these conditions involve the temporary clamping of the aorta at various levels, resulting in ischemia and subsequent tissue damage in the extremities [1]. Ischemia leads to the rapid accumulation of intracellular sodium, hydrogen, and calcium ions, resulting in tissue acidosis [2]. The lack of oxygenation for tissues shifts aerobic metabolism to anaerobic metabolism, accompanied by the increased consumption of high-energy substrates. Upon graft placement, the clamp is removed to restore blood flow in the system, initiating reperfusion. However, reperfusion produces reactive oxygen species, inflammatory mediators, and alterations in cell metabolism, exacerbating tissue damage through the formation of toxic compounds. The outcomes in tissues affected by ischemia–reperfusion injury (IRI) depend on the duration of the event, the extent of ischemic damage, and the effectiveness of the reperfusion [3].

Inflammation is critical in the pathogenesis of atherosclerosis and peripheral artery disease (PAD). In atherosclerosis, inflammatory cells infiltrate the arterial wall, leading to endothelial dysfunction, loss of glycocalyx, and the development of endotheliitis. These changes disrupt vascular homeostasis and promote plaque formation and progression [4]. Medical therapies targeting inflammation, such as statins and anti-inflammatory drugs, have effectively reduced cardiovascular events [5]. Additionally, interference with signaling pathways, like Nuclear Factor-Kappa B (NF-κB) and the heme-oxygenase pathway, can modulate inflammatory responses and improve vascular function [6].

Preconditioning, a process that involves exposing tissues to short periods of ischemia, has been shown to preserve glycocalyx integrity during subsequent ischemia–reperfusion injury [7]. This protective effect is mediated by activating the heme-oxygenase pathway, which enhances antioxidant defenses and reduces oxidative stress. Understanding these mechanisms is essential for developing therapeutic strategies to mitigate ischemia–reperfusion injury [8].

Colchicine (COL) is a microtubule-disrupting agent with anti-inflammatory and antioxidant properties used to treat various inflammatory diseases, such as acute gout, calcium pyrophosphate disease, Behçet’s syndrome, Mediterranean fever, and recurrent pericarditis [9]. It has been shown that COL reduces macrophage infiltration, cardiac remodeling, and dysfunction following IRI [10]. Similarly, the short-term administration of COL after myocardial infarction has been shown to significantly improve survival and cardiac function and reduce heart failure [11].

Mesenchymal stem cells (MSCs) are multipotent stem cells with differentiation potential in various cell types, including adipocytes, chondrocytes, osteoblasts, hepatocytes, and myoblasts, and with immunomodulatory properties. In recent years, MSCs have been used as a biological cellular approach for reducing IRI in organs [12]. This preference stems from the ease of obtaining MSCs and their relatively low tumorigenic effect. Most studies on MSCs in IRI have shown beneficial effects [13,14,15,16].

While both Colchicine and MSCs have been studied separately in IRI, there is a lack of research comparing the two in extremity IRI. Therefore, this study aims to investigate the effects of the individual and combined application of COL and MSCs on IRI in an experimental model.

## 2. Materials and Methods

### 2.1. Ethical Approval and Animal Subjects

The study was approved by the local ethics committee (approval date and number: 2020-50, 30 November 2020). The Institute of Laboratory Animals provided 42 male Wistar rats, each weighing approximately 600 g. The rats were housed in plastic cages in a controlled environment with a temperature of 23 ± 2 °C, a relative humidity of 50 ± 10%, a 12/12 light/dark cycle, and ad libitum access to food and water (~50 mL/day/rat). All subjects were male, with gender analysis revealing no significant impact.

### 2.2. Experimental Design

The rats were randomly assigned to six groups: Control (*n* = 6), receiving no treatment; Sham (*n* = 7), undergoing surgical simulation; Ischemia–Reperfusion Injury (IRI, *n* = 8), experiencing abdominal aortic ischemia; Colchicine (COL, *n* = 8), receiving 1 mg/kg colchicine intragastrically for five days pre-ischemia; Bone Marrow Mesenchymal Stem Cell (MSC, *n* = 8), receiving 300,000 MSCs intravenously immediately post-ischemia and pre-reperfusion; and Combination (Mix, *n* = 8), receiving both colchicine and MSC treatments.

### 2.3. Ischemia–Reperfusion Injury Model

General anesthesia was administered using Xylazine (5 mg/kg) and Ketamine (60 mg/kg) intraperitoneally. A median laparotomy was performed to expose the abdominal aorta, which was clamped below the renal artery using a microvascular clamp for 60 min to induce ischemia. This was followed by clamp removal to restore blood flow to both hind limbs for 120 min, as established in the relevant literature. Post-procedural euthanasia was conducted according to ethical guidelines, and blood and tissue samples were collected.

#### 2.3.1. Mesenchymal Stem Cell Preparation

MSCs were isolated from the bilateral tibias and femurs of albino Wistar rats (*n* = 9; body weight, 200–300 g) under anesthesia. Bone marrow was extracted using Roswell Park Memorial Institute (RPMI) 1640 medium (Gibco, Waltham, MA, USA) supplemented with 10% fetal bovine serum (FBS, Gibco, USA), Penicillin-Streptomycin (10,000 U/mL, Gibco, USA), and L-glutamine. The bone marrow mixture was transferred to a 50 mL falcon tube and centrifuged at 1500 rpm for 5 min. The supernatant was aspirated, and the pellet was resuspended in 5 mL of RPMI medium. This cell suspension was transferred to a 25 cm^2^ flask (Corning^®^, Corning, NY, USA) and cultured in a humidified 5% CO_2_ incubator at 37 °C. When the cells reached 80–90% confluence, they were digested with 1 mL of Trypsin-EDTA (0.25%, Gibco, USA) at 37 °C and passaged at a 1:3 ratio.

#### 2.3.2. Surface Antigen Characterization

**Figure 1:**
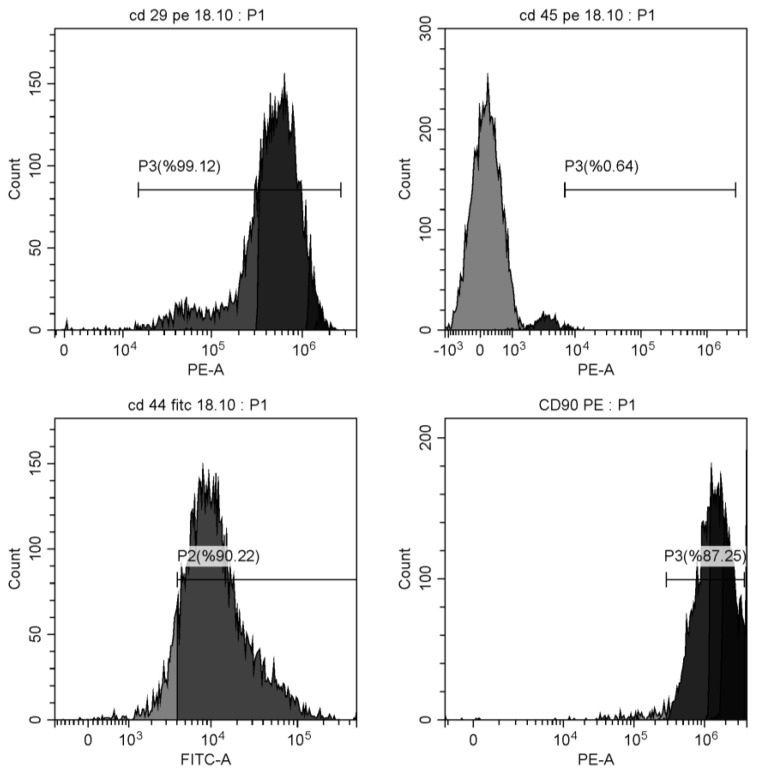
Positivity and negativity rates of cell surface antigens in obtaining bone marrow mesenchymal stem cells.

Third-generation MSCs were obtained and adjusted to a 1.2 × 10^6^ cells/mL concentration. A 100 μL cell suspension was placed into a flow cytometry tube, and 10 μL of surface antigen antibodies (Anti-CD44/FITC, Anti-CD29/PE, Anti-CD90/PE, and Anti-CD45/PC5) (BD, Franklin Lakes, NJ, USA) were added. Next, 100 μL of buffer (Gibco) was added to each tube, and the mixture was incubated at room temperature for 15 min. Following incubation, the cells were washed twice with phosphate-buffered saline (PBS) (Gibco), and 500 μL of buffer was added to each tube for flow cytometry detection (Beckman, Brea, CA, USA). The expected positivity rates for CD29/PE, CD44/FITC, and CD90/PE were over 95%, while the expected negativity rate for CD45/PC5 was below 5%. The observed positivity and negativity rates were CD29/PE 99.13%, CD44/FITC 90.24%, CD90/PE 87.25%, and CD45/PC5 0.64% (Figure 1).

### 2.4. Blood Sample Analysis

Blood samples were analyzed using spectrophotometric and chromatographic methods to assess parameters related to oxidative stress in skeletal muscle tissues, including the total antioxidant capacity (TAC), total oxidant status (TOS), and oxidative stress index (OSI), along with levels of nitric oxide (NO) metabolism products such as asymmetric dimethylarginine (ADMA), symmetric dimethylarginine (SDMA), NG-monomethyl-l-arginine (L-NMMA), arginine (ARG), citrulline (CIT), ornithine (ORN), and homoarginine (HoARG). Additionally, parameters reflecting thiol/disulfide homeostasis in blood, including native thiol (NT), total thiol (TT), dynamic disulfide (DySS), DySS/TT ratio, DySS/NT ratio, and NT/TT ratio, as well as the inflammatory parameter ischemia-modified albumin (IMA), were analyzed.

### 2.5. Tissue Sample Analysis

Skeletal muscle tissue samples were fixed in freshly prepared 4% paraformaldehyde in PBS (+4 °C) for 30 min and then stored in 30% sucrose solution at 4 °C in 0.1 M phosphate buffer (pH 7.4) for 12 h. Subsequently, the sections were embedded in a frozen medium, covered with poly-L-lysine-coated slides, and obtained using a frozen microtome at −25 °C.

Furthermore, tissue samples were analyzed using the terminal deoxynucleotidyl transferase dUTP nick end labeling (TUNEL) method to detect cellular apoptosis associated with DNA fragmentation and to assess the apoptotic ratio in the tissues. TUNEL-labeled and DAPI-stained cells were counted using ImageJ software version 1.54j (National Institutes of Health, Bethesda, MD, USA), and the apoptotic index was calculated using the formula “total apoptotic cells/total cells × 100”.

### 2.6. Statistical Analysis

The data obtained from this experimental investigation underwent analysis utilizing the SPSS Statistics software (version 29.0, Chicago, IL, USA). Given the relatively small sample sizes, non-parametric analytical approaches were favored. Group comparisons were conducted employing the Kruskal–Wallis test. Statistical significance was determined at a *p*-value threshold of <0.05. Results are reported as mean values accompanied by their respective standard deviations.

## 3. Results

The weights of the rats before and during the study were similar among the groups, indicating that they were comparable (Table 1). All subjects were able to tolerate the experimental model well.

### 3.1. Biochemical Results

Markers for oxidative stress were measured in serum samples (Table 2). These included TAC, TOS, and OSI. The TAC values in serum differed significantly among the groups (*p* < 0.001). However, the groups had no significant differences in thiol homeostasis parameters (*p* > 0.05) (Table 2). The levels of IMA (Table 3), which indicate inflammation, were significantly different among the groups (*p* = 0.020). The combined treatment group had the lowest inflammation levels, indicating that the combined therapy effectively suppressed inflammation.

Nitric oxide and arginine metabolism products, which indicate the endothelial function, vasodilation, and suppression of ROS synthesis during IRI, were measured in blood samples (Table 3). Significant differences were observed among the groups in ARG levels (*p* = 0.028), primarily driven by the IRI group. This finding suggests the IRI model was successfully established in the animal subjects. However, other parameters reflecting NO metabolism did not support this finding, so no significant differences were shown among the groups (*p* > 0.05).

### 3.2. Skeletal Muscle Tissue Results

Significant differences were observed among the muscle groups in terms of TAC levels (*p* = 0.022), primarily driven by the MSC group, indicating superior antioxidant capacity in the MSC group. In muscle samples, significant differences were observed among the groups in SDMA and CIT levels (*p* < 0.05), reflecting NO metabolism (Table 4).

### 3.3. Apoptosis Results in Skeletal Muscle

**Figure 2:**
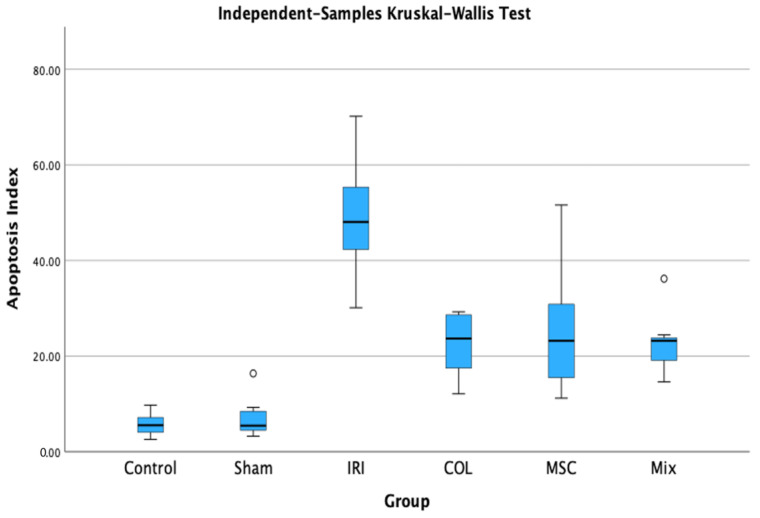
Apoptosis index comparison among different groups.

**Figure 3:**
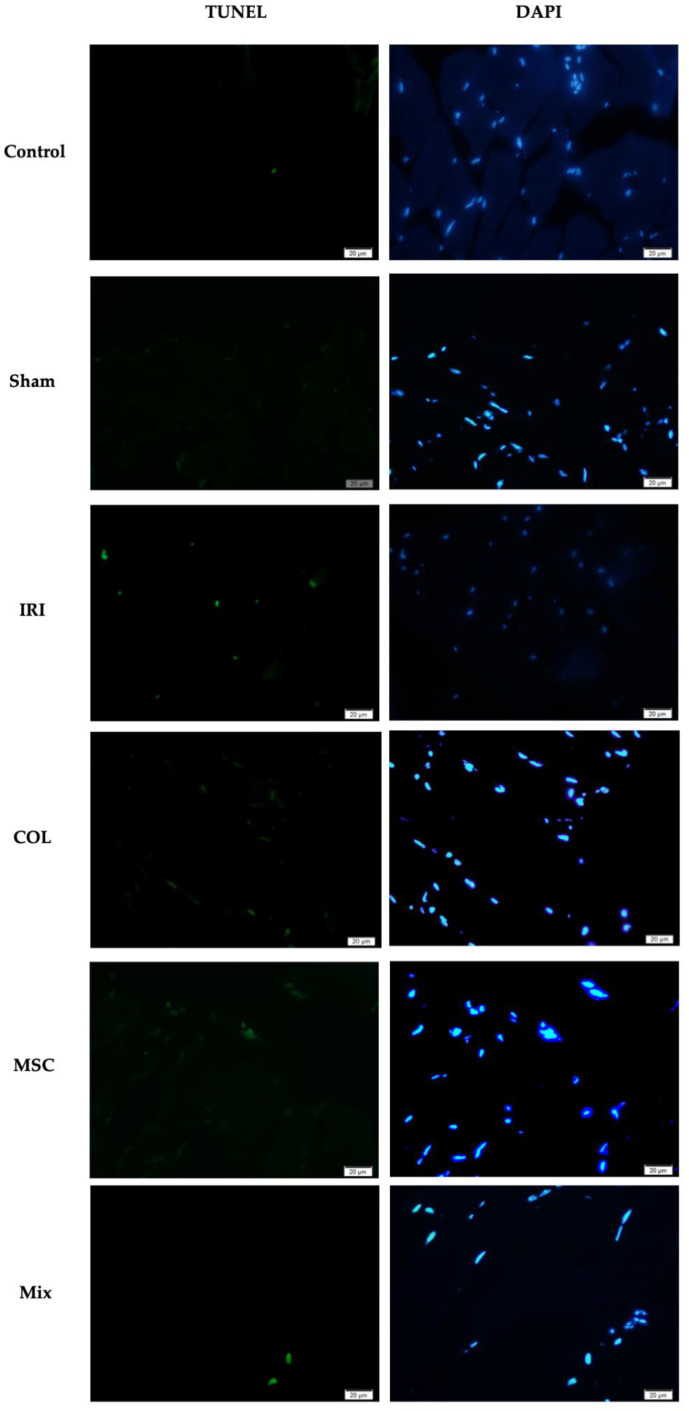
TUNEL positive cells (green) showing apoptosis in skeletal muscle sections. Cell nuclei are labeled with DAPI (blue) (40× magnification, scale bar: 20 µm).

Apoptosis in hind limb muscles was highest in the IRI group, significantly reduced by COL and MSC individually, with a more pronounced effect observed in the Mix group. The apoptotic index was highest in the IRI group (*p <* 0.001), indicating that the IRI model was successfully established (Figure 2 and Figure 3).

In summary, both COL and MSC, individually, demonstrated a tendency to attenuate skeletal muscle damage under ischemia–reperfusion conditions. Their combined use exhibited a synergistic effect, further enhancing it and demonstrating an anti-apoptotic effect by reducing cell death.

## 4. Discussion

In this study, we established a skeletal muscle IRI model in rats by subjecting them to one hour of ischemia followed by two hours of reperfusion. This model induced damage to the skeletal muscles due to IRI. Pretreatment with COL and MSC administered immediately before reperfusion individually tended to mildly reduce the damage, inflammatory response, and apoptosis in rat hind limb muscles caused by IRI. However, there was a notable trend towards enhanced effects when both treatments were combined in the Mix group.

Inflammation is crucial in the onset and progression of atherosclerosis and PAD. It triggers endothelial dysfunction and endotheliitis by disrupting the glycocalyx, facilitating plaque formation and vascular issues. Therapies, such as statins and anti-inflammatory drugs, have been shown to modulate inflammatory processes, potentially reducing cardiovascular risks. Additionally, targeting pathways, such as NF-κB and heme-oxygenase, can alleviate oxidative stress and support endothelial health, offering potential therapeutic benefits [4,5,6].

In our study, we found that COL mildly reduced IRI. However, some studies suggest that COL could significantly reduce IRI in skeletal muscles, decrease edema caused by IRI, and alleviate oxidative stress and inflammation, indicating its potential as an alternative treatment for preventing or treating skeletal muscle damage induced by IRI [17]. Additionally, COL has been found to reduce infarct size, improve hemodynamic parameters, and decrease cardiac fibrosis [10,18]. COL may reduce inflammation and oxidative stress markers, thereby alleviating liver damage caused by IRI [19]. It has also demonstrated cardioprotective and renal effects during IRI [9]. Short-term COL therapy can reduce inflammation and improve heart function, heart failure, and post-MI survival [11]. It can also be used as an adjuvant therapy to reduce early-onset ischemia–reperfusion injury and apoptosis post-surgery [20]. Furthermore, COL may reduce IRI in ovarian and testicular torsions and may protect skeletal muscle from IRI by mitigating oxidative stress and inflammation [21,22]. However, a few studies have argued against these positive findings, claiming there is no effect of COL in IRI. For example, one study found that COL did not alleviate IRI in rat intestines [23].

Our study revealed that the use of MSCs could mildly reduce IRI. The utilization of MSCs in IRI treatment dates back to the turn of the millennium. In recent years, MSCs have been employed as a biological cellular approach to mitigate IRI-related organ injuries [12]. Some researchers have compared the effectiveness of MSCs with COL treatment and investigated the impact of the timing of MSCs or COL treatment on fibrosis progression. The results demonstrated that MSCs appeared to enhance the anti-remodeling effect on the extracellular matrix more effectively than COL [24]. In another study, MSCs derived from skeletal muscle preserved kidney function following IRI [25]. Additionally, miR-143-3p, derived from MSCs, holds promising potential for IRI treatment [26]. Other studies have found that MSC-derived exosomes participate in autophagy, reducing IRI, alleviating intestinal IRI, and improving hepatic IRI [14,15,27].

Research indicates that combined therapies have been attempted to address the current issue, with findings suggesting that combined treatments can more effectively suppress IRI [28]. Our study suggests that combining COL and MSCs may be more effective in suppressing IRI. Similarly, Yin et al. argue that combined treatment with MSCs and extracorporeal shock waves is superior in treating thigh muscle IRI compared to individual applications, demonstrating the potential of combined therapy in alleviating muscle injuries [29]. Furthermore, some studies suggest that combined treatment with MSCs and extracorporeal shock waves is superior to individual use in improving critical limb ischemia [30]. Studies also claim that MSCs may act as immunomodulators in the repair of ischemic muscles [31].

Albumin is the most abundant protein in mammals. The properties of albumin change in conditions of oxidative stress, oxygen radical synthesis, and acidosis. This results in the production of ischemia-modified albumin (IMA), which is an early indicator of ischemia as it loses its cobalt-binding ability [32,33]. IMA can provide valuable information in oxidative stress conditions like ischemia–reperfusion injury (IRI). Although we expected to observe a significant increase in IMA levels in the IRI group in our study, we did not observe this. However, the combination therapy significantly reduced IMA levels compared to other groups, indicating a stronger anti-inflammatory effect.

When evaluating cellular redox status, it is crucial to consider parameters such as IRI, NT, TT, and DySS, as well as their ratios to each other. These ratios can indicate cellular redox status. Taking these parameters together can help us understand the effects of IRI on cellular redox status more comprehensively. Maintaining an appropriate redox balance is important for cellular functions [34]. However, our study did not find a statistically significant imbalance in thiol/disulfide homeostasis. This could be due to factors such as biological diversity, sensitivity of the measurement method, and sample size. Our research suggests that thiol and disulfide levels, measured at a specific time point, may vary due to dynamic processes affecting cellular redox status. Despite the lack of statistical significance, we believe our findings are biologically important when evaluated alongside other results.

In our study, we analyzed the products and markers of NO metabolism. We found that the levels of arginine in serum and SDMA and citrulline in skeletal muscle samples were statistically significant. However, we did not observe significant differences in any other parameters between the groups. After evaluating all the parameters of NO metabolism, we concluded that IRI did not affect NO metabolism in the animal model we used, possibly due to various factors. Significant levels of arginine are important for vascular regulation. On the other hand, ADMA, SDMA, and L-NMMA are substances that inhibit NO synthesis [35]. The lack of significance in these parameters suggests that there was no significant change in NO regulation among the groups in our study. Therefore, we believe that factors such as ischemia, reperfusion time, and biological factors did not affect NO synthesis in the IRI model.

Oxidative stress is a significant factor in the development of IRI affecting the muscles of the extremities. During IRI, there is an increase in the release of reactive oxygen species that can lead to inflammation and tissue damage. Previous studies have demonstrated that IRI triggers oxidative stress in skeletal muscles [36]. Our results indicate that compared to the IRI group, the levels of TOS and OSI in the serum were significantly higher in other groups, and there were significant differences among the groups in the TAC levels in the skeletal muscles. Although there was no statistically significant difference in all of the oxidative parameters, our findings suggest that the combined treatment of COL and MSCs tends to reduce oxidative stress more effectively than other treatment methods. Therefore, we believe that COL and MSCs may have partial antioxidant and anti-inflammatory effects.

The emerging evidence suggests that severe apoptosis may contribute to skeletal muscle IRI, although the precise mechanism is not yet fully understood [37,38]. This study evaluated tissue sections using TUNEL staining to assess apoptosis. We observed that the hind limb muscles in the IRI group exhibited higher levels of apoptosis compared to those in groups treated with either COL or MSCs alone. However, the combined treatment group showed significantly lower levels of apoptosis. Using COL and MSCs together might be a promising therapeutic strategy for reducing IRI-induced apoptosis.

## 5. Conclusions

This study investigated the effects of COL, MSCs, and their combination therapy on IRI-induced muscle damage. The results indicate that combination therapy is more effective in reducing oxidative stress and apoptosis than other treatments. However, further research is needed to determine the biological significance of these findings.

## Figures and Tables

**Table 1:** Comparison of pre-study and study onset weights of rats across groups.

Weight Measurement	*n*	Pre-Study (g) (Mean ± SD)	Study (g) (Mean ± SD)
Control	6	618.67 ± 39.79	614.33 ± 40.51
Sham	7	596.86 ± 53.73	604.57 ± 59.37
IRI	8	612.75 ± 41.53	612.25 ± 45.57
COL	6	602.67 ± 39.87	545.00 ± 44.81
MSC	8	598.00 ± 42.28	601.50 ± 50.24
Mix	7	596.00 ± 33.86	586.43 ± 49.95
*p*-Value		0.842	0.142

**Table 2:** Oxidative stress and thiol/disulfide homeostasis markers in blood samples.

	CONTROL (*n* = 6)	SHAM (*n* = 7)	IRI (*n* = 8)	COL (*n* = 6)	MSC (*n* = 8)	MIX (*n* = 7)	*p*-Value
	Mean ± SD	Mean ± SD	Mean ± SD	Mean ± SD	Mean ± SD	Mean ± SD	
TAC	1.47 ± 0.09	1.65 ± 0.09	1.45 ± 0.11	1.34 ± 0.09	1.38 ± 0.11	1.24 ± 0.12	0.52
TOS	6.72 ± 1.23	6.24 ± 2.91	11.17 ± 7.17	9.11 ± 3.96	9.31 ± 6.07	8.28 ± 4.26	0.039 *
OSI	4.55 ± 0.65	3.77 ± 1.72	7.61 ± 4.50	6.80 ± 2.90	6.78 ± 4.46	6.92 ± 4.15	0.000 *
NT (mmol/L)	159.67 ± 28.93	211.14 ± 111.69	254.5 ± 85.50	297.83 ± 79,71	289.63 ± 127.91	279.86 ± 124.6	0.055
TT (mmol/L)	288.5 ± 41.38	374.29 ± 211.54	475.75 ± 193.39	464.17 ± 169.36	560.75 ± 201.25	502.86 ± 183.84	0.154
DYSS (mmol/L)	64.42 ± 21.62	81.57 ± 55.53	110.63 ± 58.76	83.17 ± 46.82	135.56 ± 73.29	111.5 ± 59.32	0.337
DYSS/TT (%)	22.01 ± 5.64	21.69 ± 7.00	22.15 ± 4.42	16.67 ± 4.79	24.52 ± 9.66	22.84 ± 9.54	0.52
DYSS/NT (%)	43.00 ± 22.10	44.02 ± 27.98	42.00 ± 16.32	26.24 ± 10.29	61.96 ± 43.8	56.23 ± 49.77	0.52
NT/TT (%)	55.97 ± 11.28	56.63 ± 13.99	55.70 ± 8.85	66.66 ± 9.57	50.96 ± 19.32	54.32 ± 19.08	0.261

TAC: total antioxidant capacity, TOS: total oxidant status, OSI: oxidative stress index, NT: plasma native thiol, TT: plasma total thiol, DySS: plasma dynamic disulfide; * *p* < 0.05.

**Table 3:** Inflammatory and NO metabolism markers in blood samples.

	Control (*n* = 6)	Sham (*n* = 7)	IRI (*n* = 8)	COL (*n* = 6)	MSC (*n* = 8)	Mix (*n* = 7)	*p*-Value
	Mean ± SD	Mean ± SD	Mean ± SD	Mean ± SD	Mean ± SD	Mean ± SD	
IMA	1.43 ± 0.28	1.68 ± 0.12	1.66 ± 0.11	1.69 ± 0.11	1.65 ± 0.23	1.29 ± 0.31	0.020 *
ADMA	0.21 ± 0.15	0.16 ± 0.09	0.26 ± 0.09	0.41 ± 0.36	0.23 ± 0.09	0.3 ± 0.09	0.069
SDMA	0.40 ± 0.26	0.34 ± 0.09	0.43 ± 0.10	0.61 ± 0.42	0.40 ± 0.15	0.27 ± 0.08	0.097
L-NMMA	0.08 ± 0.06	0.07 ± 0.05	0.07 ± 0.04	0.10 ± 0.10	0.06 ± 0.04	0.06 ± 0.03	1.00
ARG	69.66 ± 13.77	54.87 ± 11.88	34.23 ± 20.88	67.53 ± 22.78	47.35 ± 20.2	58.42 ± 18.78	0.028 *
CIT	133.45 ± 67.03	131.79 ± 66.71	206.00 ± 78.48	112.15 ± 60.27	147.48 ± 63.4	103.13 ± 35.31	0.132
ORN	28.25 ± 15.16	21.35 ± 10.12	30.05 ± 7.28	21.22 ± 11.56	27.85 ± 11.16	20.51 ± 5.74	0.336
HoARG	1.42 ± 0.80	1.09 ± 0.39	1.64 ± 0.59	1.52 ± 0.74	1.36 ± 0.98	1.08 ± 0.49	0.479

IMA: ischemia-modified albumin, ADMA: asymmetric dimethylarginine, SDMA: symmetric dimethylarginine, L-NMMA: *n*-Monomethyl-L-Arginine, ARG: arginine, CIT: citrulline, ORN: ornithine, HoARG: homoarginine. * *p* < 0.05.

**Table 4:** Oxidative stress and NO markers in skeletal muscle samples.

	CONTROL (*n* = 6)	SHAM (*n* = 7)	IRI (*n* = 8)	COL (*n* = 6)	MSC (*n* = 8)	MIX (*n* = 7)	*p*-Value
	Mean ± SD	Mean ± SD	Mean ± SD	Mean ± SD	Mean ± SD	Mean ± SD	
TAC	1.01 ± 0.08	0.98 ± 0.07	0.98 ± 0.12	0.99 ± 0.14	0.83 ± 0.07	0.96 ± 0.02	0.020 *
TOS	9.86 ± 3.80	9.87 ± 3.07	11.67 ± 2.86	14.63 ± 10.59	9.74 ± 2.51	11.11 ± 0.01	0.577
OSI	9.74 ± 3.49	9.94 ± 2.58	11.76 ± 2.06	14.09 ± 7.60	11.68 ± 2.3	11.42 ± 0.01	0.499
ADMA	0.20 ± 0.10	0.18 ± 0.09	0.23 ± 0.07	0.38 ± 0.37	0.24 ± 0.06	0.39 ± 0.19	0.093
SDMA	0.10 ± 0.06	0.11 ± 0.03	0.12 ± 0.04	0.23 ± 0.17	0.07 ± 0.02	0.10 ± 0.02	0.017 *
L-NMMA	0.03 ± 0.01	0.03 ± 0.02	0.04 ± 0.03	0.04 ± 0.02	0.03 ± 0.01	0.07 ± 0.05	0.417
ARG	34.52 ± 13.31	49.27 ± 16.08	38.65 ± 17.80	41.95 ± 14.66	26.21 ± 11.21	42.54 ± 14.43	0.108
CIT	17.53 ± 11.88	19.41 ± 8.11	22.73 ± 17.69	13.70 ± 8.58	13.65 ± 3.97	37.27 ± 16.66	0.036 *
ORN	3.10 ± 1.60	3.54 ± 1.86	3.83 ± 1.52	3.58 ± 0.81	3.93 ± 1	5.19 ± 1.79	0.29
HOARG	0.20 ± 0.09	0.26 ± 0.19	0.32 ± 0.51	0.19 ± 0.08	0.25 ± 0.32	1.23 ± 1.51	0.092

TAC: total antioxidant capacity, TOS: total oxidant status, OSI: oxidative stress index, ADMA: asymmetric dimethylarginine, SDMA: symmetric dimethylarginine, L-NMMA: N-Monomethyl-L-Arginine, ARG: arginine, CIT: citrulline, ORN: ornithine, HoARG: homoarginine. * *p* < 0.05.

## Data Availability

Data from this study can be made available to interested parties upon reasonable request to the corresponding author, subject to the approval of the Ethics Board’s authorized committees.
